# Construction and Validation of a PCA-Based Prediction Model for Preterm Infant Stunting Risk: A Retrospective Study

**DOI:** 10.3390/children12050583

**Published:** 2025-04-30

**Authors:** Kun Dai, Rong Yu, Yushi Meng, Xiaomeng Yang, Yixin Jiang, Jing Luo, Kui Fang, Suqing Wang, Zhihui Rong

**Affiliations:** 1 School of Nursing, Wuhan University, Wuhan 430071, China; 2024103070003@whu.edu.cn (K.D.); 2017302280004@whu.edu.cn (Y.J.); 2024283070026@whu.edu.cn (J.L.); swang2099@whu.edu.cn (S.W.); 2 Tongji Hospital Affiliated to Tongji Medical College, Huazhong University of Science and Technology, Wuhan 430030, China; m202276415@hust.edu.cn (R.Y.); 17mengyushi@tjh.tjmu.edu.cn (Y.M.); 3 School of Public Health, Wuhan University, Wuhan 430071, China; 2022203050022@whu.edu.cn; 4 The First Affiliated Hospital of China Medical University, Shenyang 110001, China; fangkui@cmu1h.com

**Keywords:** stunting, risk factors, prediction model, principal component analysis, preterm infants

## Abstract

Objectives: Developmental delay in preterm infants is a critical clinical issue, and early risk identification and prediction are essential. This study aims to develop and validate a predictive model for developmental delay, providing a scientific basis for clinical risk assessment and early intervention. Methods: This study included preterm infants and their primary caregivers who were followed up at our center from May 2023 to September 2024. The samples were randomly divided into a training cohort, an internal validation cohort, and an external validation cohort. Independent risk factors for stunting were identified through univariate and multivariate logistic regression analyses, and predictive models and calibration were constructed accordingly. Results: The five standardized indicators at 3, 6, 9, and 12 months for 507 preterm infants were analyzed using principal component analysis, and their developmental outcomes were grouped accordingly. Logistic regression analyses showed that gestational age, high-risk factors, knowledge of caregiving, caregiving experience, and the presence of other caregivers in the home were independent risk factors for the risk of preterm infants with stunted growth at 3, 6, 9, and 12 months. The nomogram showed the area under the receiver operating characteristic curve values of 0.743, 0.735, 0.752, and 0.774 in the training cohort; 0.855, 0.771, 0.870, and 0.786 in the internal validation cohort; 0.822, 0.804, 0.717, and 0.678 in the external validation cohort, respectively. The calibration curves, consistency index, and decision curve analysis all showed that the model was significantly better than a single indicator in predicting the risk of stunting in preterm infants. Conclusions: The stunting risk prediction model constructed in this study shows good predictive ability, which can help clinicians assess the risk of stunting in preterm infants and support the development of early intervention strategies.

## 1. Introduction

According to the World Health Organization, preterm birth, defined as delivery occurring before 37 weeks of gestation, serves as one of the key indicators reflecting a nation’s health status [1,2]. A systematic analysis based on data from 103 countries and regions showed that the global incidence of preterm birth was 9.9% in 2020, with approximately 15 million births occurring annually [3]. In China, the incidence of preterm birth has steadily increased, rising from 5.36% in 1990–1994 to 6.1% in 2012–2018 [4,5]. While the continuous improvement in rescue strategies for preterm infants has led to higher survival rates, these infants continue to face numerous serious health challenges. These include conditions such as bronchopulmonary dysplasia, necrotizing enterocolitis, sepsis, and retinopathy of prematurity [6], as well as long-term health issues such as asthma, learning disorders, attention deficit disorders, emotional problems, and growth and developmental delays [7,8,9,10]. As the survival rates for preterm infants continue to rise, research attention is increasingly shifting toward their long-term growth and development [11,12].

It has been shown that early prediction and intervention can significantly reduce growth and developmental abnormalities in preterm infants [13,14]. By comprehensively analyzing maternal and infant health status, birth weight, and gestational age, high-risk preterm infants can be effectively identified and predicted, providing a scientific basis for early intervention [15]. Currently, prediction models of preterm growth and development mainly focus on neurodevelopment or somatic development [16,17]. While both neurodevelopment and somatic development are crucial to the growth process of preterm infants, many studies have not fully explored the dynamics of these two aspects or their interactions over time [18]. Furthermore, a single indicator cannot comprehensively reflect the complexity of preterm infant growth and development and the long-term trajectory. More importantly, many studies overlook the cumulative effects of multidimensional factors over time, such as neurodevelopment, socioeconomics, family environment, and quality of care, when selecting predictors [19]. These factors play a significant role in the long-term development of preterm infants, and their dynamic influence may need to be understood through longitudinal tracking data. Therefore, it is imperative to develop a prediction model for growth failure in preterm infants that integrates multiple factors such as time dimension, neurodevelopment, physical development, and socioeconomics.

Principal Component Analysis (PCA) is a widely used dimensionality reduction technique that identifies key variables based on their contributions by combining multiple related variables into a few principal components. It calculates the weights (i.e., contributions) of each principal component to form a comprehensive index [20]. The advantages of PCA include reducing data dimensionality, extracting the most important information, eliminating multicollinearity among variables, and removing redundant or non-critical information. As a result, PCA enhances the prediction accuracy and stability of the model [21]. PCA has been successfully applied across various fields, including healthcare, education, and the economy, yielding positive results [22,23].

This study aims to apply PCA to analyze the physical development indicators (such as length, weight, and head circumference) and neurodevelopmental indicators (based on the Gesell Developmental Schedules, GDS) of preterm infants to obtain the comprehensive indicators and then combine the social factors to construct a prediction model for comprehensively assessing the development of preterm infants and to overcome the limitation that a single indicator cannot comprehensively evaluate the growth and development of preterm infants. By considering multidimensional factors in the PCA analysis, we aim to build the model and explore its potential to improve prediction accuracy, providing a more reliable basis for clinical intervention and, thus, improving the long-term health prognosis of preterm infants.

## 2. Materials and Methods

### 2.1. Study Subjects

This prospective study included preterm infants who were followed up between February 2022 and March 2025 at the neonatal follow-up centers of Tongji Hospital, affiliated with Tongji Medical College of Huazhong University of Science and Technology (Hankou Hospital District and Guanggu Hospital District). The Ethics Committee approved the study protocol (WHU-LFMD-IRB2024046), and all subjects signed a written informed consent.

The inclusion criteria for preterm infants were as follows: (1) gestational age <37 weeks; (2) physical measurements and GDS measurements at four time points: 3, 6, 9, and 12 months. The exclusion criteria included (1) severe congenital anomalies, (2) inherited metabolic disorders, (3) severe asphyxia at birth, and (4) those who died or abandoned treatment.

### 2.2. Data Collection

#### 2.2.1. General Data Collection

Data collection in this study was conducted in two stages. In the first stage, clinical data on preterm infants were extracted from medical records and follow-up documentation. The collected data included infants’ sex, gestational age, birth weight, length, head circumference, Neonatal Behavioral Neurological Assessment (NBNA)—a standardized evaluation tool assessing neonatal neurobehavioral development based on neurological performance—and pregnancy-related risk factors at birth. Electronic health records were created for each preterm infant during the initial follow-up visit. In the second stage, structured questionnaires were administered to the primary caregivers of the preterm infants to gather sociodemographic and caregiving-related information. The collected data encompassed caregiver sex, age, marital status, educational attainment, employment status, place of residence, monthly household income, medical expenses, length of hospital stay, relationship with the infant, caregiving experience, caregiving-related knowledge, and the presence of other caregivers within the family.

#### 2.2.2. Outcome Data Collection

Physical growth assessment indicators included length, weight, and head circumference at corrected gestational ages of 3, 6, 9, and 12 months. Neurobehavioral development was assessed using the GDS [24], which evaluates five dimensions: adaptability, gross motor skills, fine motor skills, language, and personal–social skills. The developmental quotient (DQ) was calculated using the following formula:

$$DQ=\frac{Developmental\;Age}{Chronological\;Age\;}\times 100$$

The evaluation criteria were as follows: DQ ≥ 85 indicates normal development; 70 ≤ DQ < 85 indicates mild developmental delay; and DQ < 70 indicates severe developmental delay. Experienced professionals conducted all assessments over a one-hour session to comprehensively evaluate preterm infant development.

### 2.3. Principal Component Analysis (PCA)

This study used PCA to integrate standardized indicators of length, weight, head circumference, and the five dimensions of the GDS into a comprehensive developmental quality index, where positive values were defined as “normal development” and negative values as “developmental delay”. Based on the developmental status of preterm infants at 3, 6, 9, and 12 months, subjects were classified into “normal development” and “developmental delay” groups in the training, internal validation, and external validation cohorts to construct and validate the predictive model.

### 2.4. Development and Validation of the Nomogram

The developmental status of preterm infants at 3, 6, 9, and 12 months in the training, internal validation, and external validation sets were analyzed to compare clinical characteristic differences between groups. Subsequently, univariate analysis was conducted in each dataset to identify potential risk factors for developmental delay. Logistic regression was then used to calculate the regression coefficients for these risk factors in the training set at 3, 6, 9, and 12 months, which were incorporated into the nomogram model. The nomogram scores were further calculated in the internal and external validation sets to assess the model’s performance and applicability.

### 2.5. AUROC, Calibration Curve, C-Index, and DCA Analysis

The predictive performance of the nomogram for developmental delay at 3, 6, 9, and 12 months was validated using multiple methods. Receiver operating characteristic curves were plotted to analyze the predictive ability of the nomogram at each time point and compare it with individual indicators, with the area under the curve (AUROC) used to quantify discrimination ability. Second, the concordance index (C-index) was calculated to assess the consistency and discrimination performance of the nomogram in the training and validation sets. Calibration curves and the Hosmer–Lemeshow test were used to evaluate the agreement between the predicted and actual probabilities. Additionally, decision curve analysis (DCA) was conducted to assess the nomogram’s potential clinical value and compare it with individual indicators. These methods provided a comprehensive evaluation of the nomogram’s clinical utility.

### 2.6. Statistical Analysis

Categorical variables were presented as counts and percentages and compared using the chi-square or Fisher’s exact test. NBNA, birth weight, and birth length were described using quartiles (Q1, Q2, Q3). Length, weight, head circumference, and the five dimensions of the GDS at 3, 6, 9, and 12 months were standardized as Z-scores and analyzed using R software. PCA was conducted with a Kaiser–Meyer–Olkin (KMO) value > 0.5 and Bartlett’s test of sphericity with *p* < 0.001. Principal components were extracted based on eigenvalues and cumulative variance contribution rates (≥80%), and a linear model of the principal components was calculated using the maximum variance rotation method. A composite score was obtained by weighting the principal components according to their variance contribution rates [25]. Based on the PCA results, chi-square tests were used to compare clinical characteristics associated with developmental delay, and logistic regression was performed to calculate risk coefficients for constructing the nomogram model. All statistical tests were two-sided, with a significance level of 0.05 (* *p* < 0.05, ** *p* < 0.01, *** *p* < 0.001, **** *p* < 0.0001).

## 3. Results

### 3.1. Results of Principal Component Analysis

**Figure 1:**
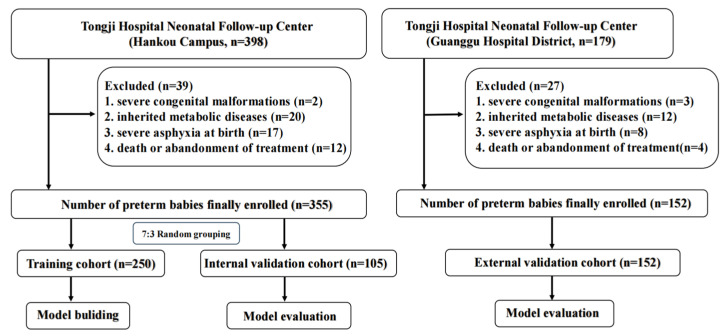
Selection strategies for preterm infants were included in this study.

The study sample included 355 preterm infants from the Hankou Hospital District and 152 preterm infants from the Guanggu Hospital District. The preterm infants from the Hankou Hospital district were randomly divided into a training cohort and an internal validation cohort in a 7:3 ratio, and the preterm infants from the Guanggu Hospital District who met the inclusion criteria were used as an external validation cohort and validated in both the internal and external validation cohorts, as shown in Figure 1.

The KMO value and Bartlett’s test of sphericity indicated that the data were appropriate for PCA (Appendix A: Table A1). In the training and internal validation cohorts, three principal components were extracted at 3 months, explaining 81.8% of the total variance; four components were extracted at 6 and 9 months, accounting for 83.8% and 82.4% of the variance, respectively, and five components were extracted at 12 months, explaining 83.8% of the total variance. Four principal components were extracted in the external validation cohorts at 3, 6, 9, and 12 months, accounting for cumulative variance contributions of 84.2%, 83.3%, 83.9%, and 81.3%, respectively. For calculating the composite developmental delay score, the outcome indicators (length, weight, head circumference, and the five dimensions of the GDS) were denoted as X_1_ to X_8_. PCA was conducted using the principal component score coefficient matrix, and a composite index formula was constructed by weighting each principal component according to its respective variance contribution rate. The total variance explained by the principal components is presented in Appendix A: Table A2; the rotated principal component score coefficients are shown in Appendix A: Table A3, and the composite index formula is provided in Appendix B.

### 3.2. General Characteristics of Preterm Infants and Primary Caregivers

In the training, internal validation, and external validation cohorts, the developmental delay group was predominantly concentrated within the gestational age range of 28–32 weeks (34.97%, 19.35%, and 45.0%, respectively), with a higher proportion of high-risk factors observed (58.74%, 32.26%, and 45.0%). The proportion of caregivers with caregiving experience was relatively low (9.79%, 4.84%, and 18.75%), as was the proportion of caregivers with caregiving knowledge (7.69%, 9.68%, and 30.0%). In contrast, the proportion of caregivers without additional support was higher (44.76%, 33.87%, and 47.5%). Educational attainment was primarily at the high school or college level (89.51%, 88.71%, and 85%, respectively). There were significant differences in these factors (*p* < 0.05) (see Appendix C).

### 3.3. Univariate Analysis of Factors Influencing Developmental Delay in Preterm Infants

**Figure 2:**
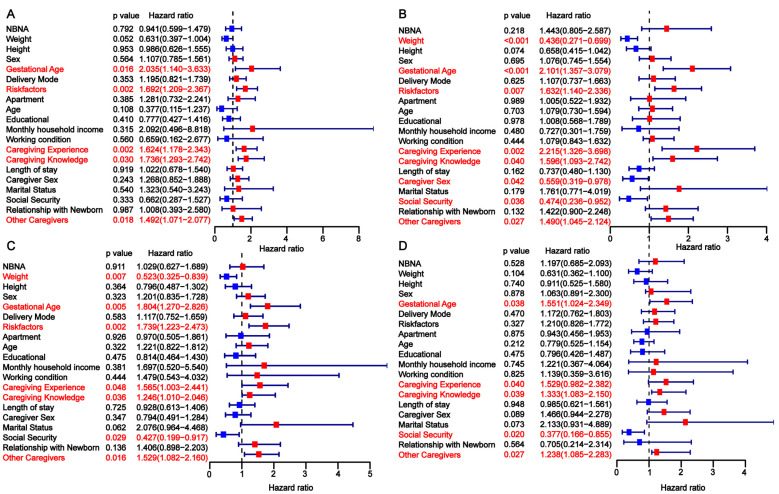
Univariate analysis of factors influencing developmental delay in preterm infants at 3 (**A**), 6 (**B**), 9 (**C**), and 12 (**D**) months in the training cohort.

**Figure 3:**
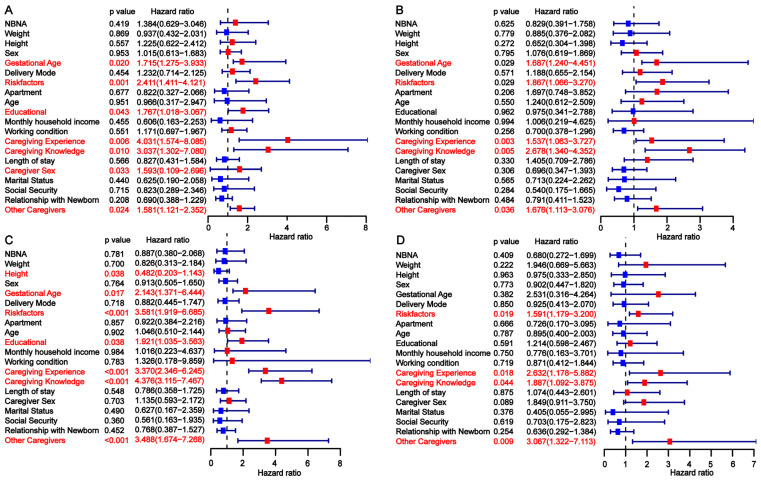
Univariate analysis of factors influencing developmental delay in preterm infants at 3 (**A**), 6 (**B**), 9 (**C**), and 12 (**D**) months in the internal validation cohort.

**Figure 4:**
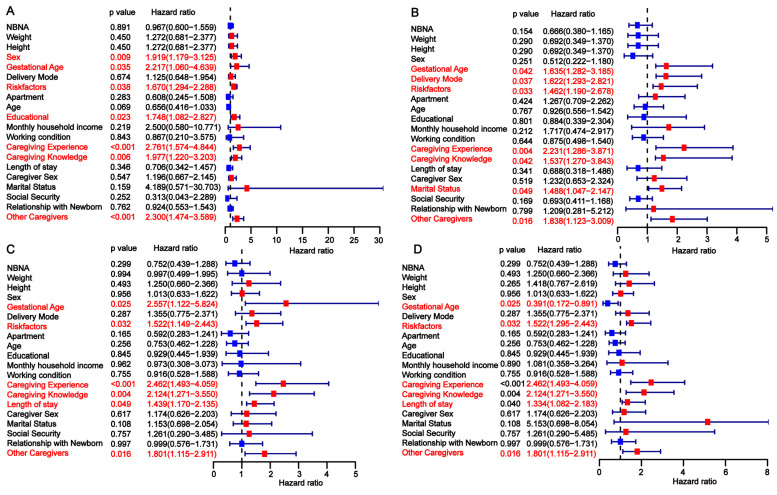
Univariate analysis of factors influencing developmental delay in preterm infants at 3 (**A**), 6 (**B**), 9 (**C**), and 12 (**D**) months in the external validation cohort.

To identify independent risk factors for developmental delay in preterm infants, we conducted a univariate analysis across the three cohorts. In the training cohort, gestational age, risk factors, primary caregiver experience, caregiver knowledge, and the presence of other caregivers were found to be significantly associated with developmental delay at 3, 6, 9, and 12 months (Figure 2). The findings from the univariate analysis in the internal validation cohort were consistent with those in the training cohort (Figure 3). In the external validation cohort, gestational age <28 weeks, the presence of perinatal risk factors, lack of caregiver knowledge and experience, and the absence of other caregivers were also identified as risk factors for developmental delay at 3, 6, 9, and 12 months (Figure 4). Overall, gestational age, risk factors, caregiver experience, caregiver knowledge, and the presence of other caregivers were significantly associated with developmental delay at all four time points.

### 3.4. Construction of the Nomogram

**Figure 5:**
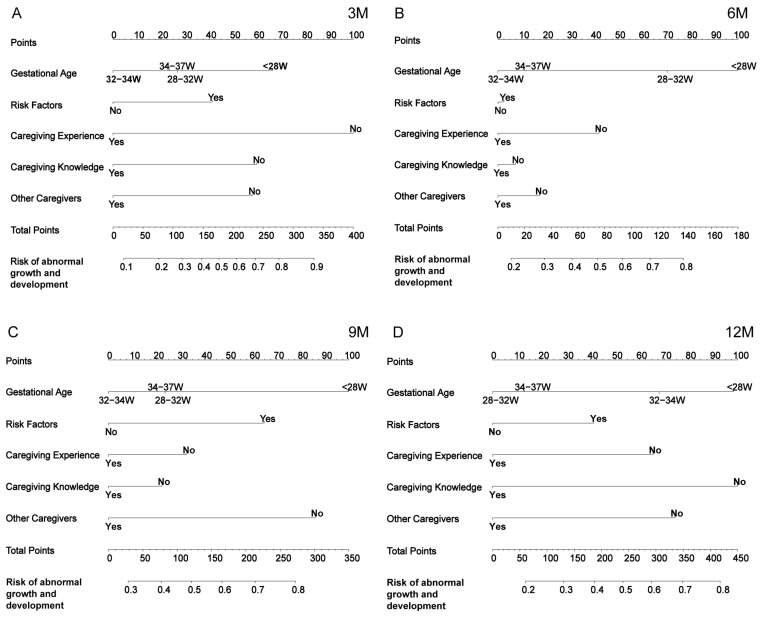
Nomogram for predicting developmental delay in preterm infants at 3 (**A**), 6 (**B**), 9 (**C**), and 12 (**D**) months.

Based on the five common independent prognostic factors identified through univariate analysis at the four time points (3, 6, 9, and 12 months), nomograms were constructed to predict a developmental delay in preterm infants at these time points. Using the nomogram, a risk score can be obtained for each preterm infant by summing the scores of each variable, thereby estimating the probability of developmental delay at 3, 6, 9, and 12 months (Figure 5).

### 3.5. Comparison Between the Nomogram and the Five Independent Factors

**Figure 6:**
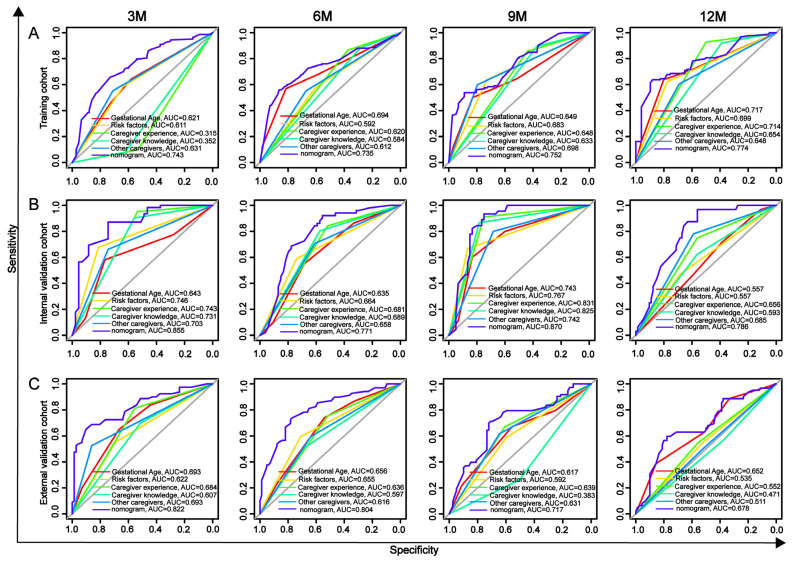
ROC curves for predicting developmental delay in preterm infants at 3, 6, 9, and 12 months. (**A**) training cohort, (**B**) internal validation cohort, and (**C**) external validation cohort.

To evaluate the predictive performance of the nomogram for developmental delay in preterm infants, ROC analysis was conducted at 3, 6, 9, and 12 months. The AUROC values of the nomogram in the training cohort were 0.743, 0.735, 0.752, and 0.774, respectively. In the internal validation cohort, the AUROC values were 0.855, 0.771, 0.870, and 0.786, and in the external validation cohort, the AUROC values were 0.822, 0.804, 0.717, and 0.678, indicating good discriminative ability. The AROUC values of the nomogram at all four time points across the three cohorts were higher than those of the five independent risk factors (gestational age, risk factors, caregiver experience, caregiver knowledge, and presence of other caregivers), demonstrating superior predictive performance (Figure 6).

### 3.6. Evaluation and Validation of the Nomogram for Predicting Developmental Delay in Preterm Infants

**Figure 7:**
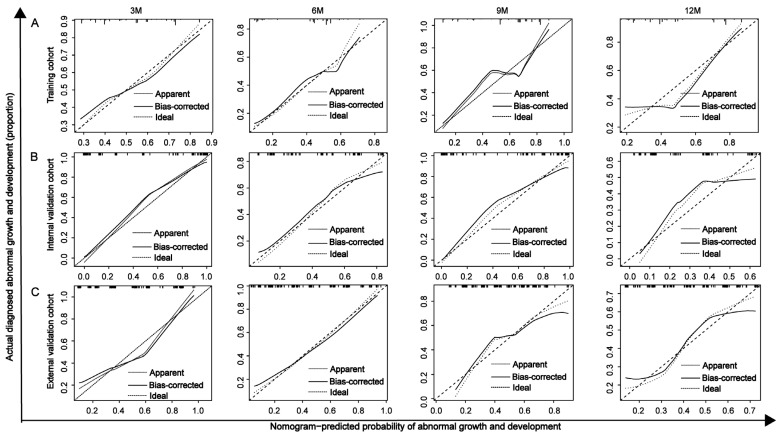
Calibration curves of the nomogram for predicting developmental delay in preterm infants at 3, 6, 9, and 12 months. (**A**) training cohort, (**B**) internal validation cohort, and (**C**) external validation cohort.

The C-index and calibration curves were used to evaluate the discrimination and reliability of the nomogram. The C-index values of the nomogram for predicting developmental delay at 3, 6, 9, and 12 months were 0.77, 0.735, 0.803, and 0.727 in the training cohort; 0.92, 0.792, 0.946, and 0.797 in the internal validation cohort; and 0.826, 0.802, 0.749, and 0.735 in the external validation cohort, demonstrating good discriminative ability. In addition, the calibration curves at all four time points across the three cohorts closely aligned with the reference line, indicating high reliability (Figure 7).

**Figure 8:**
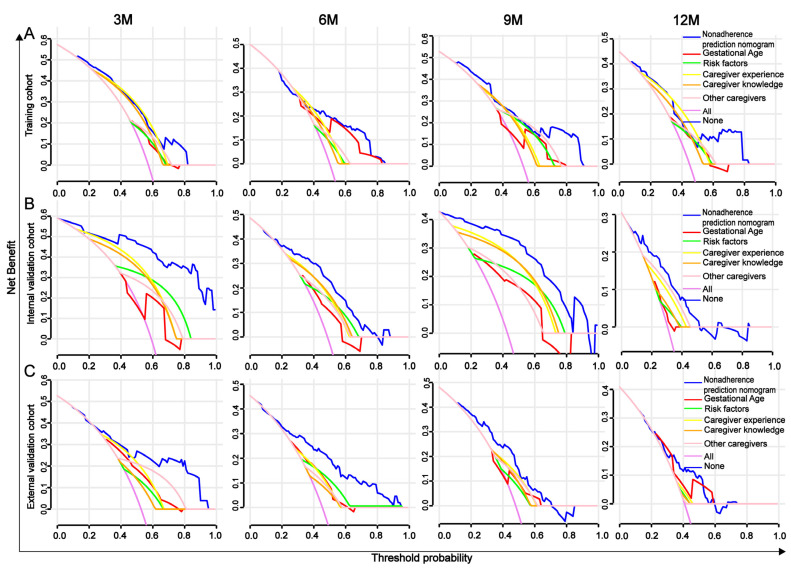
Decision curves for predicting developmental delay in preterm infants at 3, 6, 9, and 12 months. (**A**) training cohort, (**B**) internal validation cohort, and (**C**) external validation cohort.

DCA is used to assess the clinical value of diagnostic models and is considered superior to AUROC. In predicting developmental delay in preterm infants at 3, 6, 9, and 12 months, the nomogram’s DCA scores in the training, internal validation, and external validation sets were higher than those of the five independent risk factors (gestational age, risk factors, caregiver experience, caregiver knowledge, and presence of other caregivers), indicating more significant net benefit and superior clinical value (Figure 8). 

## 4. Discussion

Preterm birth is considered one of the key indicators of a country’s health status, as it is the leading cause of neonatal mortality worldwide and the second leading cause of death among children under five years of age [2]. Although significant advances in treatment strategies have improved the survival rate of preterm infants in recent years, surviving preterm infants remain at higher risk of both short-term and long-term health complications [26]. Most current studies on the prognosis of preterm infants focus on individual diseases, and comprehensive models capable of PCA were employed to integrate physical and neurodevelopmental indicators of preterm infants into a composite index, reducing data dimensionality while retaining key information. A comprehensive index was constructed based on cohort data from the Hankou and Guanggu districts of Tongji Hospital. Independent predictive factors were identified through univariate analysis and incorporated into a nomogram to predict the growth and development of preterm infants at 3, 6, 9, and 12 months. To further validate the model’s performance, ROC analysis, calibration curves, C-index, and DCA were used to compare the predictive performance of the nomogram with five independent risk factors. The results demonstrated that the nomogram exhibited superior predictive performance for growth and development at 3, 6, 9, and 12 months. These findings suggest the model could offer valuable guidance for individualized management and intervention strategies for preterm infants.

Currently, there are relatively few studies on neonatal predictive models, particularly in neonatal growth and development, where research remains limited. Existing studies primarily focus on predictive models for specific diseases. For example, Papastefanou et al. [27] used a competing risk model to analyze the joint distribution of gestational age at delivery and birth weight Z-scores, assessing the model’s predictive ability for small-for-gestational-age infants with or without preeclampsia. However, the absence of an external validation cohort limited the model’s generalizability. Tesfie et al. [28] developed a nomogram based on nine predictive factors to predict neonatal mortality, enabling individualized risk prediction. However, the lack of internal and external validation weakened the strength of the evidence. Similarly, Liu et al. [29] developed a predictive nomogram for neonatal acute respiratory distress syndrome based on five independent predictive factors, providing an effective tool for early prediction and timely treatment. However, the small sample size and absence of internal validation compromised the stability of the results. Furthermore, Cho et al. [30] explored the major risk factors for necrotizing enterocolitis in extremely low birth weight infants using six different models. However, this study relied on public database data and lacked clinical data for validation. Notably, compared with previous studies, our study offers certain advantages in data integration and model validation. By applying PCA, we integrated physical and neurodevelopmental indicators of preterm infants and combined them with clinical characteristics to develop a comprehensive predictive model. Both internal and external validation were conducted, effectively overcoming some of the limitations observed in previous research.

PCA, a commonly used dimensionality reduction tool, extracts several uncorrelated principal components through linear transformation, thereby maximizing the explained variance of the original data [31]. It has been validated across various research fields. For example, Chakraborty et al. [32] used PCA to improve the classification performance and stability of a stroke prediction model. Muhamad et al. [33] applied PCA to extract key indicators, enhancing the identification of individuals susceptible to heat stress. Deliu et al. [34] used PCA to identify key variables contributing to asthma heterogeneity, improving the model’s stability and interpretability. Zhang et al. [35] employed PCA to identify sources of heavy metal pollution in agricultural soils, providing a powerful tool for environmental monitoring. These studies demonstrate that PCA can help identify underlying patterns and improve predictive accuracy. Furthermore, the application of PCA addresses the issue of collinearity among multiple indicators [36]. Martis et al. [37] successfully applied PCA to extract features from electrocardiogram signals, enabling the automatic classification of arrhythmias and further demonstrating the practicality of PCA in the medical field. In this study, PCA was used for dimensionality reduction, and logistic regression was applied to construct the predictive model. The validation results showed that the model demonstrated good discrimination and calibration for predicting the risk of developmental delay in preterm infants at 3, 6, 9, and 12 months, providing crucial support for clinical application.

Over time, this study observed that the clinical differences in indicators (such as caregiver knowledge and caregiving ability) among the training, internal validation, and external validation sets decreased at different time points (3, 6, 9, and 12 months). This may be attributed to the greater individual variability in growth and development during the early stages of preterm infants. However, as they age, their growth curves tend to stabilize, and their physical and neurodevelopmental status gradually approaches that of full-term infants. Furthermore, social support plays a critical role in the healthy development of preterm infants. Improvements in healthcare have led to more comprehensive nutritional interventions and early treatments for preterm infants, significantly reducing the adverse effects of high-risk factors [19]. Through the follow-up system, primary caregivers received systematic education and guidance from professionals, helping them better understand the specific needs of preterm infants and implement appropriate interventions. This not only enhanced caregivers’ coping abilities but also contributed to the gradual improvement in the infants’ physical and neurodevelopmental status. The high plasticity of the preterm infant’s nervous system allows for significant mitigation of developmental impairments under appropriate interventions [38]. Long-term follow-up interventions, such as nutritional optimization, improvements in the home environment, and professional guidance, have shown significant benefits for the healthy development of preterm infants. This also explains the gradual reduction in developmental differences observed at later time points (9 and 12 months). These findings emphasize the critical role of social factors, such as accessibility to healthcare resources, the timeliness of educational efforts, and the effectiveness of family support in promoting the healthy development of preterm infants and facilitating early biological interventions. This provides a theoretical basis for the future exploration of personalized interventions and offers practical guidance for developing a more comprehensive health management system for preterm infants.

Although this study has yielded significant findings, several limitations must be considered. First, this study was based on single-center data, and the geographic specificity of the sample may limit the model’s generalizability. Future studies should incorporate multi-center data to validate the model’s stability. Second, the follow-up period in this study was relatively short, covering only the developmental status of preterm infants at 3, 6, 9, and 12 months. Longer follow-up periods are necessary to assess the model’s long-term predictive performance. Moreover, this study did not incorporate internationally recognized neurodevelopmental assessment tools such as the General Movements Assessment (GMA) and the Hammersmith Infant Neurological Examination (HINE), which have demonstrated high sensitivity and feasibility for early prediction of cerebral palsy and related disorders [39]. According to guidelines by Novak et al. [40], GMA combined with MRI is recommended before 5 months of corrected age, and HINE with MRI after 5 months. However, this study did not follow these standards or benchmark its indicators against established gold standards, potentially limiting the model’s clinical applicability and scientific validity. Despite these limitations, this study innovatively combined physical and neurodevelopmental indicators with social factors to develop a comprehensive predictive model based on clinical and social characteristics. This model demonstrated good predictive performance at multiple time points, providing a scientific foundation for the individualized management of preterm infants. Additionally, this study highlighted the importance of educating primary caregivers and enhancing their caregiving abilities during early interventions, which was shown to play a key role in improving the growth and development of preterm infants. These findings offer valuable insights for optimizing intervention strategies and developing long-term care plans for preterm infants.

## 5. Conclusions

This study developed a predictive model for the growth and developmental risks of preterm infants based on physical and neurodevelopmental indicators using PCA. The model’s predictive performance at 3, 6, 9, and 12 months was validated, demonstrating high discrimination and calibration ability, thus providing a scientific foundation for individualized management and intervention strategies for preterm infants. This study emphasizes the importance of early intervention in improving preterm infant developmental outcomes. Despite limitations related to the geographic specificity of the sample and the relatively short follow-up period, the findings offer both theoretical support and practical guidance for optimizing the health management of preterm infants.

## Data Availability

The data presented in this study are available upon reasonable request from the corresponding author. Due to privacy and ethical restrictions, certain data cannot be publicly shared.

## Abbreviations

The following abbreviations are used in this manuscript:

PCA	Principal Component Analysis
GDS	Gesell Developmental Schedules
NBNA	Neonatal Behavioral Neurological Assessment
DQ	The developmental quotient
AUROC	The area under the curve
C-index	The concordance index
DCA	Decision curve analysis
KMO	Kaiser–Meyer–Olkin

## Appendix A

**Table A1:** Results of the KMO Value and Bartlett’s Test of Sphericity.

Month	Training and Internal Validation			External Validation		
	KMO	Bartlett’s Test	*p*	KMO	Bartlett’s Test	*p*
March	0.820	1639.571	<0.001	0.740	507.794	<0.001
June	0.810	1308.35	<0.001	0.800	568.103	<0.001
September	0.830	1217.548	<0.001	0.750	518.131	<0.001
December	0.790	662.763	<0.001	0.810	439.026	<0.001

**Table A2:** Total Variance Explained.

Time Point	Ingredients	Test Set and Internal Validation			External Verification		
		Initial Eigenvalue	Variance Contribution Rate (%)	Cumulative Variance Contribution Rate (%)	Initial Eigenvalue	Variance Contribution Rate (%)	Cumulative Variance Contribution Rate (%)
3M	1	0.001	0.479	0.479	1.656	0.343	0.343
	2	1.332	0.222	0.701	1.545	0.298	0.641
	3	0.97	0.118	0.818	0.986	0.121	0.763
	4	0.712	0.063	0.881	0.799	0.08	0.842
	5	0.567	0.04	0.922	0.666	0.055	0.898
	6	0.501	0.031	0.953	0.584	0.043	0.941
	7	0.446	0.025	0.978	0.552	0.038	0.979
	8	0.42	0.022	1	0.414	0.021	1
6M	1	1.879	0.441	0.441	1.867	0.436	0.436
	2	1.427	0.254	0.696	1.471	0.271	0.706
	3	0.812	0.083	0.778	0.742	0.069	0.775
	4	0.691	0.06	0.838	0.681	0.058	0.833
	5	0.624	0.049	0.886	0.662	0.055	0.888
	6	0.603	0.045	0.932	0.615	0.047	0.935
	7	0.541	0.037	0.968	0.538	0.036	0.971
	8	0.502	0.032	1	0.48	0.029	1
9M	1	1.879	0.441	0.441	1.86	0.433	0.433
	2	1.387	0.241	0.682	1.325	0.22	0.652
	3	0.805	0.081	0.763	0.97	0.118	0.77
	4	0.696	0.061	0.824	0.746	0.07	0.839
	5	0.647	0.052	0.876	0.671	0.056	0.895
	6	0.641	0.051	0.927	0.585	0.043	0.938
	7	0.569	0.041	0.968	0.508	0.032	0.971
	8	0.507	0.032	1	0.485	0.029	1
12M	1	1.688	0.356	0.356	1.864	0.434	0.434
	2	1.176	0.173	0.529	1.149	0.165	0.599
	3	0.943	0.111	0.64	1.008	0.127	0.726
	4	0.912	0.104	0.744	0.834	0.087	0.813
	5	0.867	0.094	0.838	0.739	0.068	0.882
	6	0.747	0.07	0.908	0.625	0.049	0.93
	7	0.693	0.06	0.968	0.585	0.043	0.973
	8	0.506	0.032	1	0.464	0.027	1

**Table A3:** Rotated Principal Component Score Coefficients.

Time Point	Indicators	Test Set and Internal Validation					External Verification			
		F1	F2	F3	F4	F5	F1	F2	F3	F4
3M	X _1_	−0.011	0.562	−0.152			0.060	0.367	−0.096	−0.103
	X _2_	−0.022	−0.072	0.994			−0.018	0.378	0.01	0.055
	X _3_	−0.025	0.527	0.036			−0.071	0.357	0.061	0.04
	X _4_	0.242	−0.012	−0.002			0.281	−0.021	−0.025	0.179
	X _5_	0.203	0.005	−0.09			−0.046	−0.013	0.999	−0.032
	X _6_	0.231	−0.034	0.007			−0.229	−0.003	−0.025	1.038
	X _7_	0.232	0.015	0.028			0.423	0.008	−0.064	−0.128
	X _8_	0.239	−0.022	0.015			0.569	−0.017	0.003	−0.412
6M	X _1_	0.019	−0.214	1.057	−0.044		−0.142	0.367	0.441	−0.193
	X _2_	0.015	0.487	0.037	−0.043		0.025	0.397	0.04	−0.132
	X _3_	−0.052	0.695	−0.364	0.039		0.095	0.38	−0.625	0.268
	X _4_	0.472	−0.015	−0.031	−0.373		0.546	0.022	−0.238	−0.296
	X _5_	0.312	−0.03	0.077	−0.087		0.362	0.023	0.282	−0.362
	X _6_	0.481	0.009	−0.016	−0.391		0.462	−0.058	−0.355	0.01
	X _7_	0.074	−0.015	0.038	0.374		−0.281	−0.045	−0.105	1.179
	X _8_	−0.324	0.004	−0.058	1.114		−0.119	−0.044	0.956	0.035
9M	X _1_	0.001	−0.216	1.064	0.082		0.132	0.478	0.187	−0.197
	X _2_	0.168	0.52	−0.002	−0.459		−0.147	0.643	−0.277	0.175
	X _3_	−0.125	0.692	−0.314	0.36		−0.085	−0.11	0.904	0.019
	X _4_	0.221	−0.031	0.089	0.102		−0.361	−0.019	0.095	0.718
	X _5_	−0.141	0.01	0.031	1.083		0.721	−0.045	−0.45	−0.428
	X _6_	0.233	−0.039	0.092	0.079		−0.156	0.021	−0.138	0.544
	X _7_	0.377	0.093	−0.121	−0.352		0.467	0.016	−0.078	−0.136
	X _8_	0.414	−0.013	−0.017	−0.446		0.258	0.001	0.052	0.094
12M	X _1_	−0.013	−0.093	−0.016	−0.1	1.024	−0.002	1.019	−0.093	−0.114
	X _2_	−0.004	−0.067	0.021	1.019	−0.1	−0.023	−0.1	1.012	0.026
	X _3_	−0.049	1.019	0.03	−0.066	−0.092	−0.017	−0.107	0.02	0.972
	X _4_	−0.115	0.03	1.03	0.021	−0.02	0.226	−0.083	−0.044	0.143
	X _5_	0.319	0.01	−0.141	0.082	−0.11	0.221	−0.022	0.008	0.072
	X _6_	0.267	−0.13	0.057	−0.023	0.06	0.258	−0.001	−0.021	−0.055
	X _7_	0.336	−0.007	−0.077	−0.014	0.041	0.243	−0.058	0.03	0.029
	X _8_	0.337	0.017	−0.08	−0.053	−0.012	0.254	0.168	−0.02	−0.228

## Appendix B

3M Training and Internal Validation Set: Principal Component Score Formula: F1 = −0.011X_1_ − 0.022X_2_ − 0.025X_3_ + 0.242X_4_ + 0.203X_5_ + 0.231X_6_ + 0.232X_7_ + 0.239X_8_; F2 = 0.562X_1_ − 0.072X_2_ + 0.527X_3_ − 0.012X_4_ + 0.005X_5_ − 0.036X_6_ + 0.015X_7_ − 0.022X_8_; F3 = −0.152X_1_ + 0.994X_2_ + 0.036X_3_ − 0.002X_4_ − 0.090X_5_ + 0.007X_6_ + 0.028X_7_ + 0.015X_8_. Composite Index = 0.475F1 + 0.216F2 + 0.127F3.

3M External Validation Set: Principal Component Score Formula: F1 = 0.060X_1_ − 0.018X_2_ − 0.071X_3_ + 0.281X_4_ − 0.046X_5_ − 0.229X_6_ + 0.423X_7_ + 0.569X_8_; F2 = 0.367X_1_ + 0.378X_2_ + 0.357X_3_ − 0.021X_4_ − 0.013X_5_ − 0.003X_6_ + 0.008X_7_ − 0.017X_8_; F3 = −0.096X_1_ + 0.010X_2_ + 0.061X_3_ − 0.025X_4_ + 0.999X_5_ − 0.025X_6_ − 0.064X_7_ + 0.003X_8_; F4 = −0.103X_1_ + 0.055X_2_ + 0.040X_3_ + 0.179X_4_ − 0.032X_5_ + 1.038X_6_ − 0.128X_7_ − 0.412X_8_. Composite Index = 0.309*F1* + 0.274*F2* + 0.126*F3* + 0.133*F4*.

6M Training and Internal Validation Set: Principal Component Score Formula: F1 = 0.019X_1_ + 0.015X_2_ − 0.052 X_3_ + 0.472X_4_ + 0.312X_5_ + 0.481X_6_ + 0.074X_7_ − 0.324X_8_; F2 = −0.214X_1_ + 0.487X_2_ + 0.695X_3_ − 0.015X_4_ − 0.030X_5_ + 0.009X_6_ − 0.015X_7_ + 0.004X_8_; F3 = 1.057X_1_ + 0.037X_2_ − 0.364X_3_ − 0.031X_4_ + 0.077X_5_ − 0.016X_6_ + 0.038X_7_ − 0.058X_8_; F4 = −0.044X_1_ − 0.043X_2_ + 0.039X_3_ − 0.373X_4_ − 0.087X_5_ − 0.391X_6_ + 0.374X_7_ + 1.114X_8_. Composite Index = 0.358*F1* + 0.211*F2* + 0.128*F3* + 0.141*F4*.

6M External Validation Set: Principal Component Score Formula: F1 = −0.142X_1_ + 0.025X_2_ + 0.095 X_3_ + 0.546X_4_ + 0.362X_5_ + 0.462X_6_ − 0.281X_7_ − 0.119X_8_; F2 = 0.367X_1_ + 0.397 X_2_ + 0.380X_3_ + 0.022X_4_ + 0.023X_5_ − 0.058X_6_ − 0.045X_7_ − 0.044X_8_; F3 = 0.441 X_1_ + 0.040X_2_ − 0.625X_3_ − 0.238X_4_ + 0.282X_5_ − 0.355X_6_ − 0.105X_7_ + 0.956X_8_; F4 = −0.193X_1_ − 0.132X_2_ + 0.268X_3_ − 0.296X_4_ − 0.362X_5_ + 0.010X_6_ + 1.179X_7_ + 0.035X_8_. Composite Index = 0.307*F1* + 0.292*F2* + 0.106*F3* + 0.128*F4*.

9M Training and Internal Validation Set: Principal Component Score Formula: F1 = 0.001X_1_ + 0.168X_2_ − 0.125X_3_ + 0.221X_4_ − 0.141X_5_ + 0.233X_6_ + 0.377X_7_ + 0.414X_8_; F2 = −0.216X_1_ + 0.520X_2_ + 0.692X_3_ − 0.031X_4_ + 0.010X_5_ − 0.039X_6_ + 0.093X_7_ − 0.013X_8_; F3 = 1.064X_1_ − 0.002X_2_ − 0.314X_3_ + 0.089X_4_ + 0.031X_5_ + 0.092X_6_ − 0.121X_7_ − 0.017X_8_; F4 = 0.082X_1_ − 0.459X_2_ + 0.360X_3_ + 0.102X_4_ + 1.083X_5_ + 0.079X_6_ − 0.352X_7_ − 0.446X_8_. Composite Index = 0.397*F1* + 0.197*F2* + 0.127*F3* + 0.103*F4*.

9M External Validation Set: Principal Component Score Formula: F1 = 0.132X_1_ − 0.147X_2_ − 0.085X_3_ − 0.361X_4_ + 0.721X_5_ − 0.156X_6_ + 0.467X_7_ + 0.258X_8_; F2 = 0.478X_1_ + 0.643X_2_ − 0.110X_3_ − 0.019X_4_ − 0.045X_5_ + 0.021X_6_ + 0.016X_7_ + 0.001X_8_; F3 = 0.187X_1_ − 0.277X_2_ + 0.904X_3_ + 0.095X_4_ − 0.022X_5_ − 0.138X_6_ − 0.078X_7_ + 0.052X_8_; F4 = −0.197X_1_ + 0.175X_2_ + 0.019X_3_ + 0.718X_4_ − 0.428X_5_ + 0.544X_6_ − 0.136X_7_ + 0.094X_8_. Composite Index = 0.258*F1* + 0.203*F2* + 0.141*F3* + 0.236*F4*.

12M Training and Internal Validation Set: Principal Component Score Formula: F1 = 0.019X_1_ + 0.015X_2_ − 0.052X_3_ + 0.472X_4_ + 0.312X_5_ + 0.481X_6_ + 0.074X_7_ − 0.324X_8_; F2 = −0.214X_1_ + 0.487X_2_ + 0.695X_3_ − 0.015X_4_ − 0.030X_5_ + 0.009X_6_ − 0.015X_7_ + 0.004X_8_; F3 = 1.057X_1_ + 0.037X_2_ − 0.364X_3_ − 0.031X_4_ + 0.077X_5_ − 0.016X_6_ + 0.038X_7_ − 0.058X_8_; F4 = −0.044X_1_ − 0.043X_2_ + 0.039X_3_ − 0.373X_4_ − 0.087X_5_ − 0.391X_6_ + 0.374X_7_ + 1.114X_8_. Composite Index = 0.358*F1* + 0.211*F2* + 0.128*F3* + 0.141*F4*.

12M External Validation Set: Principal Component Score Formula: F1 = −0.002X_1_ − 0.023X_2_ − 0.017X_3_ + 0.226 X_4_ + 0.221X_5_ + 0.258X_6_ + 0.243X_7_ + 0.254X_8_; F2 = 1.019X_1_ − 0.100X_2_ − 0.107X_3_ − 0.083X_4_ − 0.022X_5_ − 0.001X_6_ − 0.058X_7_ + 0.168X_8_; F3 = −0.093X_1_ + 1.012X_2_ + 0.020X_3_ − 0.044X_4_ + 0.008X_5_ − 0.021X_6_ + 0.030X_7_ − 0.020X_8_; F4 = −0.114X_1_ + 0.026X_2_ + 0.972X_3_ + 0.143X_4_ + 0.072X_5_ − 0.055X_6_ + 0.029X_7_ − 0.228X_8_. Composite Index = 0.433*F1* + 0.126*F2* + 0.125*F3* + 0.129*F4.*

## Appendix C

General Demographic Characteristics

		**Training Cohort**			**Internal Validation Cohort**			**External Validation Cohort**		
**Characteristics**		**Healthy** **Development**	**Developmental** **Impairment**	***p***	**HealthyDevelopment**	**DevelopmentalImpairment**	***p***	**Healthy** **Development**	**Developmental** **Impairment**	***p***
		**N = 107 (%)**	**N = 143 (%)**		**N = 43 (%)**	**N = 62 (%)**		**N = 72 (%)**	**N = 80 (%)**	
Premature infant										
NBNA	High	16 (14.95)	26 (18.18)	0.706	8 (18.60)	9 (14.52)	0.7255	36 (50.00)	43 (53.75)	0.2584
	Middle	32 (29.91)	45 (31.47)		14 (32.56)	18 (29.03)		21 (29.17)	28 (35.00)	
	Low	59 (55.14)	72 (50.35)		21 (48.84)	35 (56.45)		15 (20.83)	9 (11.25)	
Sex	Female	37 (34.58)	56 (39.16)	0.5423	21 (48.84)	33 (53.23)	0.8073	39 (54.17)	23 (28.75)	0.0025
	Male	70 (65.42)	87 (60.84)		22 (51.16)	29 (46.77)		33 (45.83)	57 (71.25)	
Gestational Age	<28 W	8 (7.48)	8 (5.59)	0.0073	4 (9.30)	8 (12.90)	0.0016	5 (6.94)	16 (20.00)	0.0003
	28–32 W	24 (22.43)	52 (36.36)		6 (13.95)	28 (45.16)		19 (26.39)	36 (45.00)	
	32–34 W	16 (14.95)	33 (23.08)		21 (48.84)	12 (19.35)		16 (22.22)	15 (18.75)	
	34–37 W	59 (55.14)	50 (34.97)		12 (27.91)	14 (22.58)		32 (44.44)	13 (16.25)	
Delivery Mode	Cesarean Section	84 (78.50)	105 (73.43)	0.4376	34 (79.07)	43 (69.35)	0.3775	57 (79.17)	64 (80.00)	1
	Natural birth	23 (21.50)	38 (26.57)		9 (20.93)	19 (30.65)		15 (20.83)	16 (20.00)	
Risk Factors	No	68 (63.55)	59 (41.26)	0.0008	35 (81.40)	20 (32.26)	<0.0001	50 (69.44)	36 (45.00)	0.0041
	Yes	39 (36.45)	84 (58.74)		8 (18.60)	42 (67.74)		22 (30.56)	44 (55.00)	
Primary caregivers										
Apartment	City	80 (74.77)	112 (78.32)	0.6593	31 (72.09)	51 (82.26)	0.3869	65 (90.28)	71 (88.75)	0.4191
	Towns	10 (9.35)	14 (9.79)		4 (9.30)	5 (8.06)		6 (8.33)	5 (6.25)	
	Villages	17 (15.89)	17 (11.89)		8 (18.60)	6 (9.68)		1 (1.39)	4 (5.00)	
Caregivers Age	<20 Y	31 (28.97)	44 (30.77)	0.7018	7 (16.28)	16 (25.81)	0.2779	18 (25.00)	33 (41.25)	0.1167
	20–30 Y	71 (66.36)	94 (65.73)		34 (79.07)	42 (67.74)		49 (68.06)	43 (53.75)	
	31–40 Y	5 (4.67)	4 (2.80)		1 (2.33)	4 (6.45)		5 (6.94)	3 (3.75)	
	41–50 Y	0 (0)	1 (0.70)		1 (2.33)	0 (0.00)		0 (0.00)	1 (1.25)	
Educational level	Primary and lower	4 (3.74)	2 (1.40)	0.0004	2 (4.65)	0 (0.00)	0.008	1 (1.39)	4 (5.00)	0.0003
	Middle or high school	88 (82.24)	128 (89.51)		38 (88.37)	55 (88.71)		56 (77.78)	68 (85.00)	
	College and above	15 (14.02)	13 (9.09)		3 (6.98)	7 (11.29)		15 (20.83)	8 (10.00)	
Monthly Household Income	<2 K	5 (4.67)	2 (1.40)	0.0472	0 (0.00)	3 (4.84)	0.0572	5 (6.94)	2 (2.50)	0.6123
	2–6 K	29 (27.10)	47 (32.87)		13 (30.23)	23 (37.10)		13 (18.06)	14 (17.50)	
	7–10 K	40 (37.38)	67 (46.85)		15 (34.88)	27 (43.55)		36 (50.00)	44 (55.00)	
	>10 K	33 (30.84)	27 (18.88)		15 (34.88)	9 (14.52)		18 (25.00)	20 (25.00)	
Working Condition	Employee	79 (73.83)	102 (71.33)	0.4641	33 (75.00)	36 (58.06)	0.1397	56 (77.78)	62 (77.5)	0.8474
	Unemployed	28 (26.17)	41 (28.67)		10 (22.73)	26 (41.94)		16 (22.22)	18 (22.50)	
Caregiving Experience	No	79 (73.83)	127 (88.81)	0.0036	20 (46.51)	59 (95.16)	<0.0001	32 (44.44)	65 (81.25)	<0.0001
	Yes	28 (26.17)	16 (11.19)		23 (53.49)	3 (4.84)		40 (55.56)	15 (18.75)	
Caregiving Knowledge	No	76 (71.03)	129 (90.21)	0.0002	19 (44.19)	56 (90.32)	<0.0001	35 (48.61)	56 (70.00)	<0.0001
	Yes	31 (28.97)	14 (9.79)		24 (55.81)	6 (9.68)		37 (51.39)	24 (30.00)	
Length of Stay	<14 D	39 (36.45)	41 (28.67)	0.4142	9 (20.93)	22 (35.48)	0.1818	52 (72.22)	52 (65.00)	0.6253
	14–23 D	34 (31.78)	49 (34.27)		16 (37.21)	23 (37.10)		14 (19.44)	19 (23.75)	
	>23 D	34 (31.78)	53 (37.06)		18 (41.86)	17 (27.42)		6 (8.33)	9 (11.25)	
Caregiver Sex	Female	91 (85.05)	111 (77.62)	0.1894	35 (81.40)	40 (64.52)	0.0963	60 (83.33)	66 (82.50)	1
	Male	16 (14.95)	32 (22.38)		8 (18.60)	22 (35.48)		12 (16.67)	14 (17.50)	
Marital Status	Married or Cohabiting	102 (95.3)	138 (96.50)	0.8859	40 (93.02)	62 (100.00)	0.1299	72 (100.00)	79 (98.75)	1
	Single	5 (4.67)	5 (3.50)		3 (6.98)	0 (0.00)		0 (0.00)	1 (1.25)	
Insurance payment	Commercial Insurance	7 (6.54)	6 (4.20)	0.133	2 (4.65)	5 (8.06)	0.4469	0 (0.00)	1 (1.25)	0.3177
	Rural Medical Care	18 (16.82)	36 (25.17)		6 (13.95)	13 (20.97)		55 (76.39)	56 (70.00)	
	Self-pay	9 (8.41)	20 (13.99)		8 (18.60)	6 (9.68)		12 (16.67)	11 (13.75)	
	Social Insurance	73 (68.22)	81 (56.64)		27 (62.79)	38 (61.29)		5 (6.94)	12 (15.00)	
Relationship with Newborn	Father	25 (23.36)	36 (25.17)	0.7037	9 (20.93)	16 (25.81)	0.8021	21 (29.17)	20 (25.00)	0.5874
	Mother	76 (71.03)	102 (71.33)		33 (76.74)	44 (70.97)		50 (69.44	57 (71.25)	
	Grandparent	6 (5.61)	5 (3.50)		1 (2.33)	2 (3.23)		1 (1.39)	3 (3.75)	
Other Caregivers	No	23 (21.50)	79 (55.24)	<0.0001	11 (25.58)	41 (66.13)	<0.0001	10 (13.89)	42 (52.50)	<0.0001
	Yes	84 (78.50)	64 (44.76)		32 (74.42)	21 (33.87)		62 (86.11)	38 (47.50)	
